# Toner’s Sketch of Early American Medicine

**Published:** 1875-01

**Authors:** 


Contributions to the Annals of Medical Progress and Medical Education in the United States, before and during the War of Independence. By Joseph M. Toner, M.D. Washington: Government Printing Office. 1874. Octavo, pamphlet, pp. 118.

We have been greatly interested in reading this retrospect of the early medical history of our country from the pen of Dr. Toner. It is full of information which cannot fail to interest and profitably instruct every American physician who would form a just estimate of the labors of the noble band of pioneers who laid deeply and surely the broad and solid foundation of the American Medicine of our day of which we may justly be proud. The pamphlet, we presume, is to be obtained by application to the Bureau of Education of the Department of the Interior, at Washington.

---

Clinical Ureametry. By Henry G. Piffard, M.D., Surgeon to the Charity Hospital, Clinical Professor of Dermatology, Medical Department of the New York University. New York: D. Appleton & Co. 1874. Octavo, pamphlet, pp. 7.

